# A comparative evaluation of the effect of polymer chemistry and fiber orientation on mesenchymal stem cell differentiation

**DOI:** 10.1002/jbm.a.35829

**Published:** 2016-07-20

**Authors:** David C.L. Rowland, Thomas Aquilina, Andrei Klein, Osnat Hakimi, Pierre Alexis‐Mouthuy, Andrew J Carr, Sarah J.B. Snelling

**Affiliations:** ^1^ Nuffield Department of Orthopaedics, Rheumatology and Musculoskeletal SciencesUniversity of OxfordOxfordUnited Kingdom

**Keywords:** electrospinning, PDO, PLGA, mesenchymal stem cells

## Abstract

Bioengineered tissue scaffolds in combination with cells hold great promise for tissue regeneration. The aim of this study was to determine how the chemistry and fiber orientation of engineered scaffolds affect the differentiation of mesenchymal stem cells (MSCs). Adipogenic, chondrogenic, and osteogenic differentiation on aligned and randomly orientated electrospun scaffolds of Poly (lactic‐co‐glycolic) acid (PLGA) and Polydioxanone (PDO) were compared. MSCs were seeded onto scaffolds and cultured for 14 days under adipogenic‐, chondrogenic‐, or osteogenic‐inducing conditions. Cell viability was assessed by alamarBlue metabolic activity assays and gene expression was determined by qRT‐PCR. Cell‐scaffold interactions were visualized using fluorescence and scanning electron microscopy. Cells grew in response to scaffold fiber orientation and cell viability, cell coverage, and gene expression analysis showed that PDO supports greater multilineage differentiation of MSCs. An aligned PDO scaffold supports highest adipogenic and osteogenic differentiation whereas fiber orientation did not have a consistent effect on chondrogenesis. Electrospun scaffolds, selected on the basis of fiber chemistry and alignment parameters could provide great therapeutic potential for restoration of fat, cartilage, and bone tissue. This study supports the continued investigation of an electrospun PDO scaffold for tissue repair and regeneration and highlights the potential of optimizing fiber orientation for improved utility. © 2016 The Authors Journal of Biomedical Materials Research Part A Published by Wiley Periodicals, Inc. J Biomed Mater Res Part A: 104A: 2843–2853, 2016.

## INTRODUCTION

Tissue engineering aims to repair and regenerate tissues or organs, eliminating the need for transplantation and mechanical devices.^1^ The development of bioengineered tissue scaffolds to be used in combination with cells and/or growth factors holds a great promise for tissue repair. These biomimetic scaffolds with properties similar to that of the extracellular matrix (ECM) allow cells to grow and differentiate along a desired cell lineage. Acting as an ECM analogue, the scaffold supplies a niche for cell growth and differentiation. The ideal biomimetic scaffold is biodegradable, non‐immunogenic, and has a porosity that allows the diffusion of nutrients and clearance of waste products.^2^ Moreover, the scaffold should support cell viability, proliferation, differentiation, and ECM production. Finally, it should have adequate mechanical properties matching the target tissue.

MSCs are often used in tissue engineering due to their ease of amplification and purification, as well as their multipotency allowing differentiation down adipogenic, chondrogenic, and osteogenic lineages.^3^, ^4^, ^5^ Scaffolds that facilitate each of these differentiation paths are sought for various applications. Scaffolds that support adipogenesis are required for the restoration of soft tissue defects such as congenital deformities, posttraumatic repair, and cancer. Scaffolds that facilitate chondrogenesis would be desirable for the restoration of cartilage following traumatic and pathological damage to articular joints, whilst those that facilitate osteogenesis could provide an alternative to the traditional bone graft techniques.^6^ Current allograft and autograft techniques for the repair of these tissue types are inadequate, with poor long‐term outcomes and carry significant complications.^7^, ^8^, ^9^, ^10^

Electrospinning is an attractive scaffold fabrication technique as it may be used to create biomimetic scaffolds that are highly instructive to cells. Electrospun fibers have the inherent advantage of a high surface area to volume ratio with nanoscale topography similar to ECM.^11^ It has also been demonstrated that electrospun materials cause a lower immune response *in vivo* compare to the same material in plain sheets.^12^ Previous studies have shown that electrospun scaffolds made of polymers such as PLGA and Polydioxanone (PDO) exhibit excellent cellular response and biocompatibility.^13^, ^14^ It has also been reported that multilineage differentiation into osteoblasts, adipocytes, and chondrocytes is fully supported by an electrospun tissue scaffold.^15^

Electrospun PLGA scaffolds have previously been shown to support successful differentiation of mesenchymal stem cells (MSCs) *in vitro* for the generation of bone, cartilage and dermal tissue.^16^, ^17^, ^18^ Studies of *in vitro* MSC differentiation on electrospun PDO are limited. Electrospun PDO scaffolds have shown to facilitate growth of human dental pulp stem cells and differentiation of adipose‐derived stem cells down adipogenic and chondrogenic cell lines *in vitro*.^19^, ^20^ *In vivo* studies have shown that electrospun PDO scaffolds facilitate the differentiation of MSCs into vascular tissue.^21^

The aim of this comparative study was to determine the effect of the chemical and physical properties of electrospun scaffolds on MSC behavior, specifically looking at the extent of cell growth and differentiation of MSCs into adipogenic, chondrogenic, and osteogenic lineages. To do so, two distinct synthetic polymers showing promise in tissue engineering were compared: PDO and PLGA. While electrospun, each of these polymers was processed into either an aligned or random orientation that reflect the gross organization of the ECM. Our aim was to identify the polymer and fiber orientation that best facilitates MSC differentiation down the adipogenic, chondrogenic, and osteogenic lineages.

## MATERIALS AND METHODS

### Cell scaffold preparation

Polymers, PDO (Sigma‐Aldrich, Gillingham, Dorset) and PLGA (75% lactic, 25% glycolic) (Sigma‐Aldrich), were dissolved in 1,1,1,3,3,3‐Hexafluro‐2‐propanol (HFP, Fluka Analytical/Sigma‐Aldrich). Polymer solutions were prepared and voltage was applied as summarized in Table 1. Polymers were electrospun using a single nozzle setup (Glassman, Bramley, Hampshire). The polymer solution was supplied with a syringe pump (Harvard apparatus‐PHD 2000, Kent) deposited on an aluminum foil at a constant flow rate of 1 mL/hour, producing a scaffold at approximately 2 cm^2^/hour. The drum was rotated at 2000 rpm for the production of aligned fibers and at 100 rpm for the production of randomly oriented fibers. The four resulting scaffold types were named as: PDO aligned (PDOa), PDO random (PDOr), PLGA aligned (PLGAa), and PLGA random (PLGAr).

**Table 1:** Comparison of Polymer Concentration, Voltage Applied, and Subsequent Fiber Diameter for PLGA and PDO Scaffolds

	PLGA		PDO	
Polymer concentration (wt % in solution)	16		9	
Voltage applied (kV)	7–8		8–9.6	
	Aligned	Random	Aligned	Random
Fiber diameter ± SD	1.36 μm ± 0.34	1.70 μm ± 0.19	1.22 μm ± 0.54	1.87 μm ± 0.42

For cell seeding, scaffolds were cut into 2 cm^2^ squares and suspended using CellCrown^TM^ six‐well plate inserts (Sigma‐Aldrich, Gillingham, Dorset). The suspended scaffolds were sterilized in 70% ethanol for 2 hours, dried for 12 hours at 40°C, and then transferred to six‐well plates (Corning, Corning, NY).

### Mesenchymal stem cells

Primary human MSCs from three donors (Lonza, Cologne, Germany) were individually expanded to a maximum of passage 10 in MesenPRO RS™ Medium (MesenPRO RS™ Basal Medium and MesenPRO RS™ Growth Supplement), with 2 m*M* glutamine and 100 IU/mL penicillin and 100 mg/mL streptomycin (Life technologies Ltd, Paisley). This basal growth medium was refreshed every 2–3 days.

### Seeding cells on scaffolds

Eighteen CellCrown inserts (Sigma Aldrich) were prepared for each primary MSC donor for each scaffold type (PDOa, PDOr, PLGAa, and PLGAr) and placed in six‐well plates. Scaffolds were conditioned in 2 mL basal medium for 1 hour prior to seeding. About 1 mL MSCs at 2 × 10^5^ cells/mL in basal media were then incubated on each scaffold for 1 hour before addition of 1 mL of basal growth media. Seeded scaffolds were incubated for a further 12 hours to allow cell attachment.

### Cell differentiation

For each scaffold type, three cell inserts per MSC donor repeat were induced (i) down the adipogenic, osteogenic, or chondrogenic cell lineage and three cell inserts remained non‐induced (c) in respective non‐inducing media for 14 days. Therefore, for each MSC donor, three intra experiment repeats were carried out for each scaffold type in each differentiation condition. Media was refreshed every 2–3 days.

For adipogenesis, the non‐inducing basal medium comprised of Dulbecco's Modified Eagle's Medium DMEM (Life technologies Ltd) containing 10% fetal bovine serum (FBS) and 100 IU/mL penicillin and 100 mg/mL streptomycin. The adipogenic medium comprised of StemPro^®^ adipocyte differentiation basal medium and StemPro^®^ adipogenesis supplement (Life technologies Ltd) 100 IU/mL penicillin and 100 mg/mL streptomycin. For osteogenesis, the non‐inducing basal medium comprised of Dulbecco's Modified Eagle's Medium DMEM containing 15% fetal calf serum (FCS), 100 IU/mL penicillin and 100 mg/mL streptomycin. The osteogenic inducing medium comprised of basal medium plus, 10 m*M* beta glycerophosphate, 50 µg/mL ascorbate‐2‐phosphate, and 10 n*M* dexamethasone. For chondrogenesis, the non‐inducing basal medium comprised of Dulbecco's Modified Eagle's Medium DMEM containing 2 m*M* glutamine and 100 IU/mL penicillin and 100 mg/mL streptomycin . The chondrogenic inducing medium comprised of basal medium, 1% insulin/transferrin/selenium/linoleic Acid (BD Biosciences, Oxford), 40 µg/mL ascorbate‐2‐phosphate (Sigma‐Aldrich), 100 n*M* Dexamethasone (Sigma‐Aldrich), and 10 ng/mL TGFβ3 (R&D Systems, Abingdon).

### Visualization of cell attachment and morphology

#### Scanning electron microscope (SEM) imaging

At day 14 one scaffold from each condition was removed and divided between SEM and fluorescence imaging. For SEM the scaffold was fixed overnight in glutaraldehyde (2.5% v/v in deionized water) before undergoing sequential dehydration in a graded ethanol series, further dehydration using hexamethyldisilazane and drying overnight. Dehydrated scaffolds were mounted on an aluminum stub using a carbon adhesive disk and gold coated using a SC7620 Mini Sputter Coater System (Quorum Technologies Ltd, East Sussex). High‐resolution images were taken using an environmental scanning electron microscope (ESEM, Carl Zeiss Evo LS15 Variable Pressure Scanning Electron Microscope). Three different areas selected randomly were observed on each sample with three different magnifications (400×, 1000×, and 5000×). Cell coverage at day 14 was calculated using ImageJ by manually drawing around cells and calculating the relative surface area occupied. fiber diameter and pore area were calculated using ImageJ. For fiber diameter 15 fibers per SEM image were measured using ImageJ and the average diameter calculated, and mean fiber diameter for *n* = 3 scaffolds of each type was calculated. For pore area the image was flattened and binarised before manually drawing around 15 pores per image and calculating average pore area, mean pore area for *n* = 3 scaffolds of each type was calculated.

#### Fluorescence imaging

Scaffolds were fixed in 10% formalin for 10 min and permeabilized using 0.1% Triton‐X (Sigma‐Aldrich) for 5 min. Cells were stained for 30 min in Alexa Fluor^®^ 488 Phalloidin (Phalloidin 2 mg/mL) with 4′,6‐diamidino‐2‐phenylindole (DAPI 2 mg/mL) nuclear counter stain according to manufacturer's instructions (Life Technologies). Scaffolds were visualized using a fluorescence microscope with a 10× objective (Zeiss Axio Imager 2, Jena, Germany).

### Determination of cell viability

The alamarBlue viability assay was carried out on days 1, 7, and 14 after seeding on all cell‐seeded scaffolds (i.e., for each of the three MSC donors three intra‐experimental repeats were carried out per scaffold for each differentiation condition). Medium was removed and the cell inserts transferred into fresh well plates containing 5% alamarBlue (abD serotec, Oxford, in non‐inducing medium). After 2 hours of incubation, duplicate samples were taken from each well for analysis of fluorescence at excitation 530 nm emission 585 nm in a FLUOstar Optima (BMG Labtech, Aylesbury). The remaining alamarBlue solution was removed and replaced with the appropriate inducing or non‐inducing medium. Viability of the cells on each scaffold at day 7 and day 14 was normalized to the viability for that specific scaffold at day 1.

### Gene expression

At day 14 the scaffolds (*n* = 2 intra‐experimental repeats for *n* = 3 MSC donors) were removed from CellCrown inserts and homogenized in Trizol (Life Technologies) using the GentleMacs dissociator (Miltenyi Biotec Ltd). RNA was extracted, cDNA synthesized and RT‐PCR was carried out according to the manufacturers protocol (Roche, Welwyn Garden City).

SybrGreen Real‐time qPCR (Life Technologies) was carried out using commercially available primers (Qiagen) for peroxisome proliferator‐activated receptor gamma (*PPARG*), fatty acid‐binding protein (*FABP*), SRY (sex determining region Y)‐box 9 (*SOX9*), Aggrecan (*ACAN*), Runt‐relate transcription factor 2 (*RUNX*2), and bone gamma‐carboxyglutamate acid containing protein (*BGLAP*).

Expression of genes of interest was normalized against the endogenous control *GAPDH*. The Veriti^®^ 96‐Well Fast Thermal Cycler was used for cDNA synthesis, and the ViiA™ 7 Real‐Time PCR System (Life Technologies) was used for analysis of gene expression. Gene expression was calculated as arbitrary units of mRNA relative to *GAPDH* reference gene using the 2^−ΔΔCT^ method.

### Statistical analysis

Results are presented in the form of mean ± standard deviation, with *n* equal to the number of biological repeats (*n* = 3 MSC donors). For the alamarBlue assays the mean of duplicate samples for each of the three intra‐experimental repeats per MSC donor was calculated and used to calculate a mean viability for the relevant condition. The overall mean viability for *n* = 3 donors was calculated from the intra‐experimental mean and used for statistical analyses. For qPCR the mean of two technical pipetting repeats per condition was used to calculate the mean expression for the two intra‐experimental repeats per MSC donor. The mean expression for each condition for each MSC donor was then used to calculate the overall mean expression (*n* = 3 donors). Two‐way analysis of variance (ANOVA) was performed to determine the effects of scaffold type on cell viability. One‐way ANOVA with Tukey's post‐test was performed to determine assess fiber diameter, pore size, cell coverage, and the effects of scaffold type on gene expression. All analyses were performed using GraphPad Prism. *p* < 0.05 was considered statistically significant. *≤0.05, **≤0.01, ***≤0.001, and ****≤0.0001.

## RESULTS

### Scaffold characteristics, cell morphology, and cell coverage

**Figure 1:**
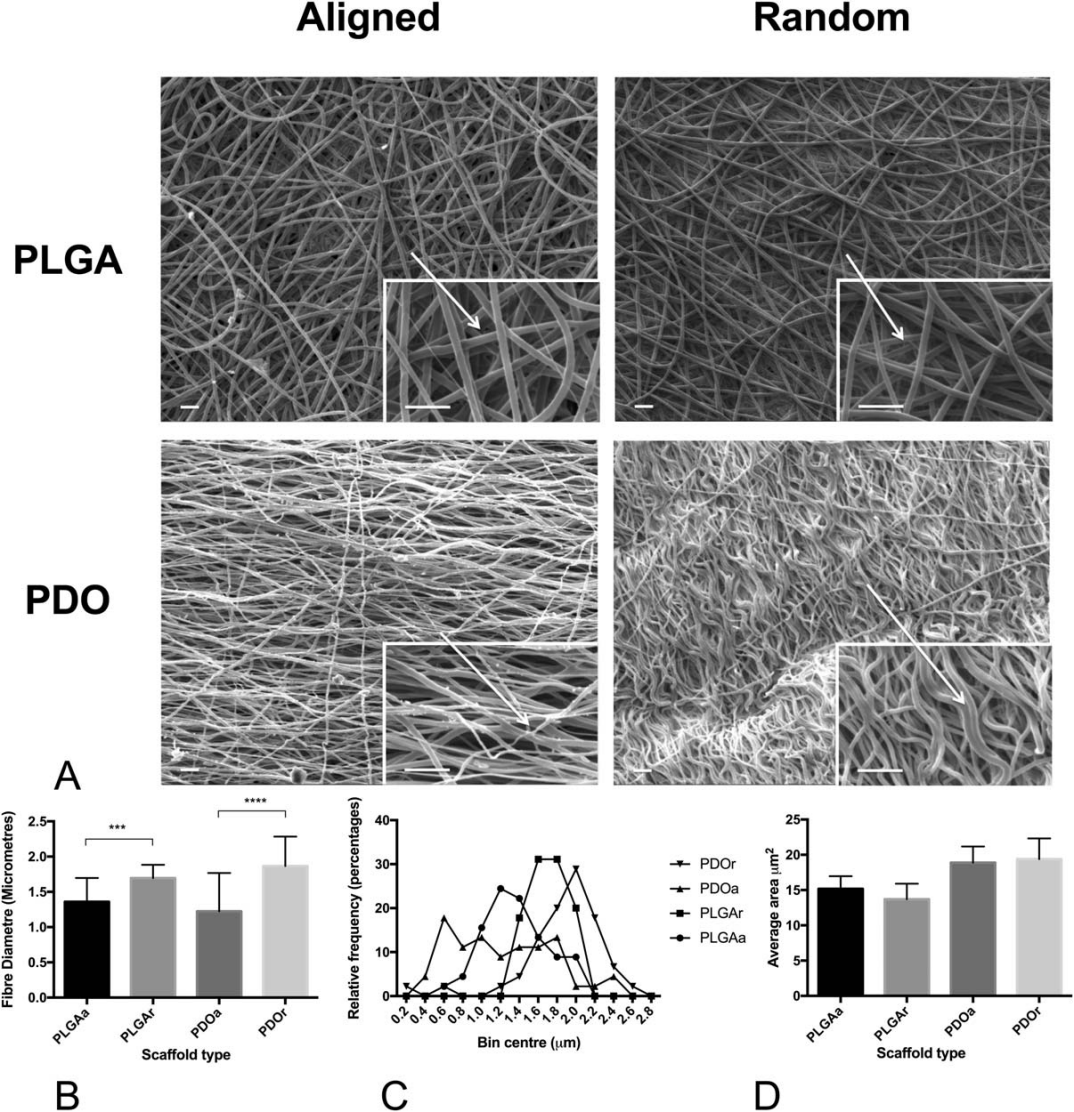
Characteristics of aligned and random PLGA and PDO scaffolds. (A) SEM of cell free scaffolds at 1000× magnification (scale bar = 10 μm) with a 5000× magnification image (scale bar = 2 μm) in bottom right hand corner. (B) Average diameter of fibers in each scaffold type. (C) Average area of pores in each scaffold type.

SEM images of scaffolds prior to cell seeding show the characteristic microarchitecture of PLGA and PDO in aligned and random orientations (Fig. 1). Aligned PLGA fibers formed in a crimp‐like fashion. Fiber diameter of PLGAr was 1.3‐fold (*p* < 0.0001) higher than PLGAa and that of PDOr was 1.5‐fold (*p* < 0.0001) higher than PDOa.

**Figure 2:**
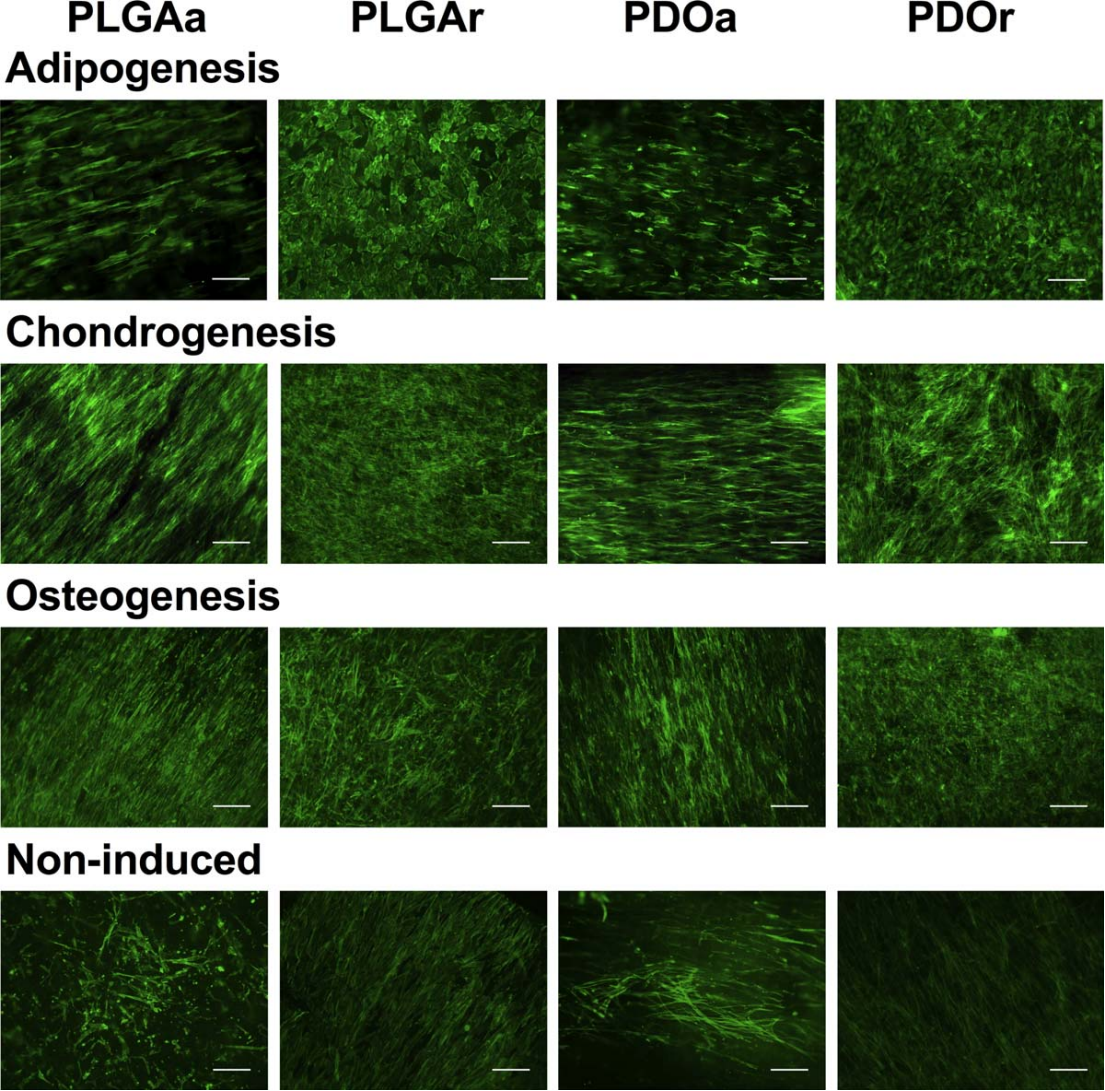
MSCs attached and grew in response to the nanopattern of the scaffold. Fluorescence microscopy images of non‐induced and adipogenic, chondrogenic and osteogenic MSCs on scaffolds at day 14. Green = phalloidin. Scale bar shows 200 µm.

**Figure 3:**
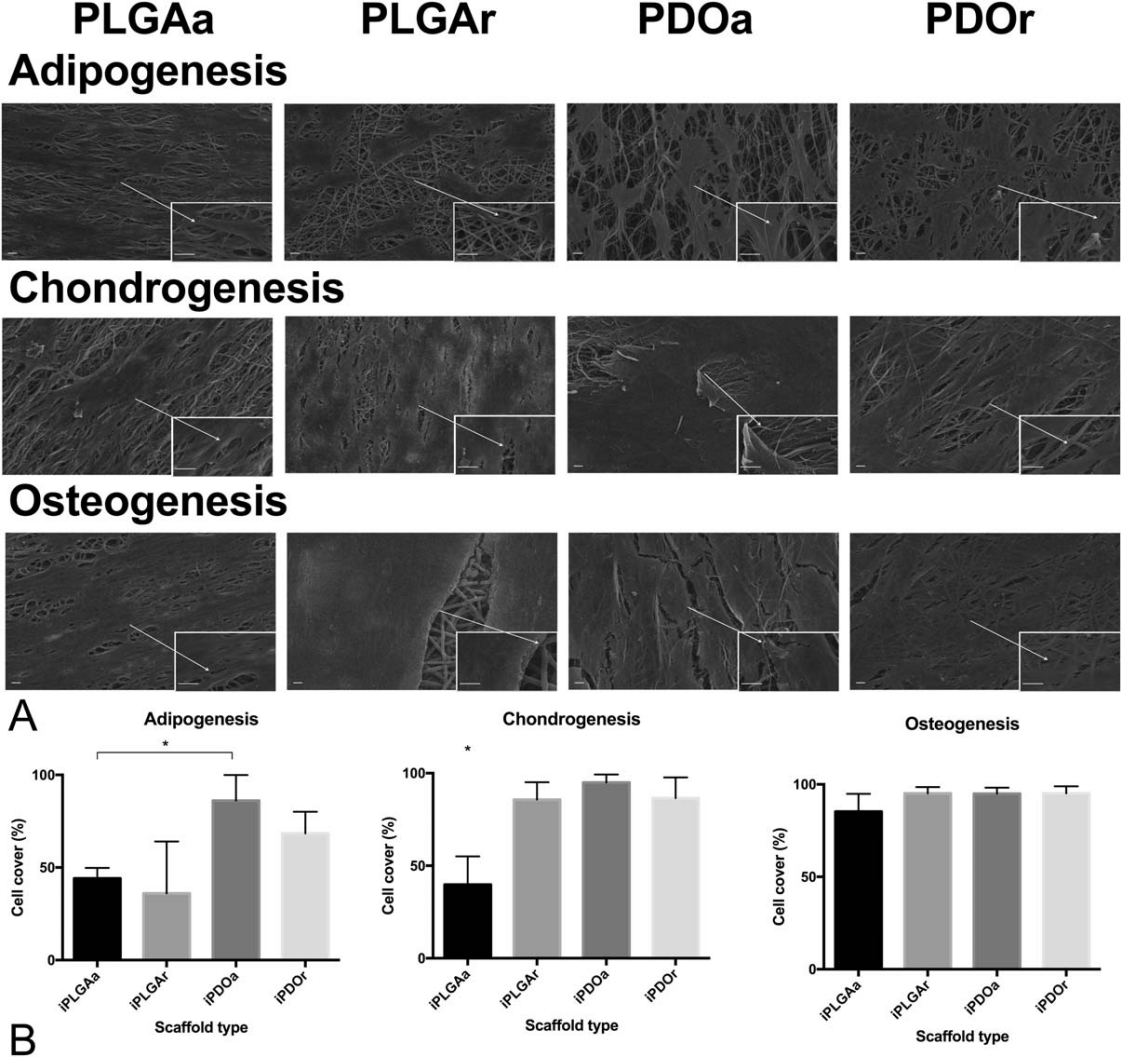
Higher adipogenic and chondrogenic cell coverage on PDO scaffolds. (A) SEM images of each scaffold type at a 1000× magnification (scale bar = 10 μm) with a 5000× magnification image (scale bar = 2 μm) in bottom right hand corner showing cells at day 14 of culture in adipogenic, chondrogenic, and osteogenic medium. The percentage cell coverage at day 14 of culture on each scaffold type was calculated from SEM images for cells in (B) adipogenic, chondrogenic, osteogenic inducing medium. *n* = 3 MSC donors.

Fluorescence microscopy and SEM images of MSCs cultured in adipogenic, osteogenic, and chondrogenic inducing medium showed good cell attachment, with cells growing in response to the fiber orientation of the scaffold (Figs. 2 and 3). Cells induced to undergo differentiation showed coverage of all scaffolds with networks of interconnected cells. On aligned scaffolds, cells grew mostly along the fiber long axis. In contrast, many of the cells grown on a random fiber orientation were polygonal in shape and randomly distributed in accordance with the fiber orientation, in particular those grown in chondrogenic and adipogenic media. Moreover, cells cultured in chondrogenic medium had visibly higher nodule formation on PDOr compared with PDOa (Fig. 2). Cells cultured in adipogenic medium appeared to have a rounder morphology on PLGAr compared with PDOr.

In terms of cell coverage, cells cultured in adipogenic inducing medium covered an area that was 2.39‐fold higher on aligned PDO relative to random PLGA [*p* < 0.05; Fig. 3(B)]. Cells coverage in chondrogenic medium was greater than twofold lower on aligned PLGA compared with all other scaffold types [*p* < 0.05; Fig. 2(B)] whilst coverage was unchanged across all scaffold types during osteogenic differentiation [Fig. 3(B)].

MSCs, cultured in non‐inducing medium, typically grew with the spindle morphology of a fibroblast‐like cell with morphology similar to MSCs maintained in a monolayer culture. When cultured on a randomly oriented fibers, these non‐differentiated cells formed relatively aligned dense monolayers of spindle shaped cells (Fig. 2).

### Cell viability

**Figure 4:**
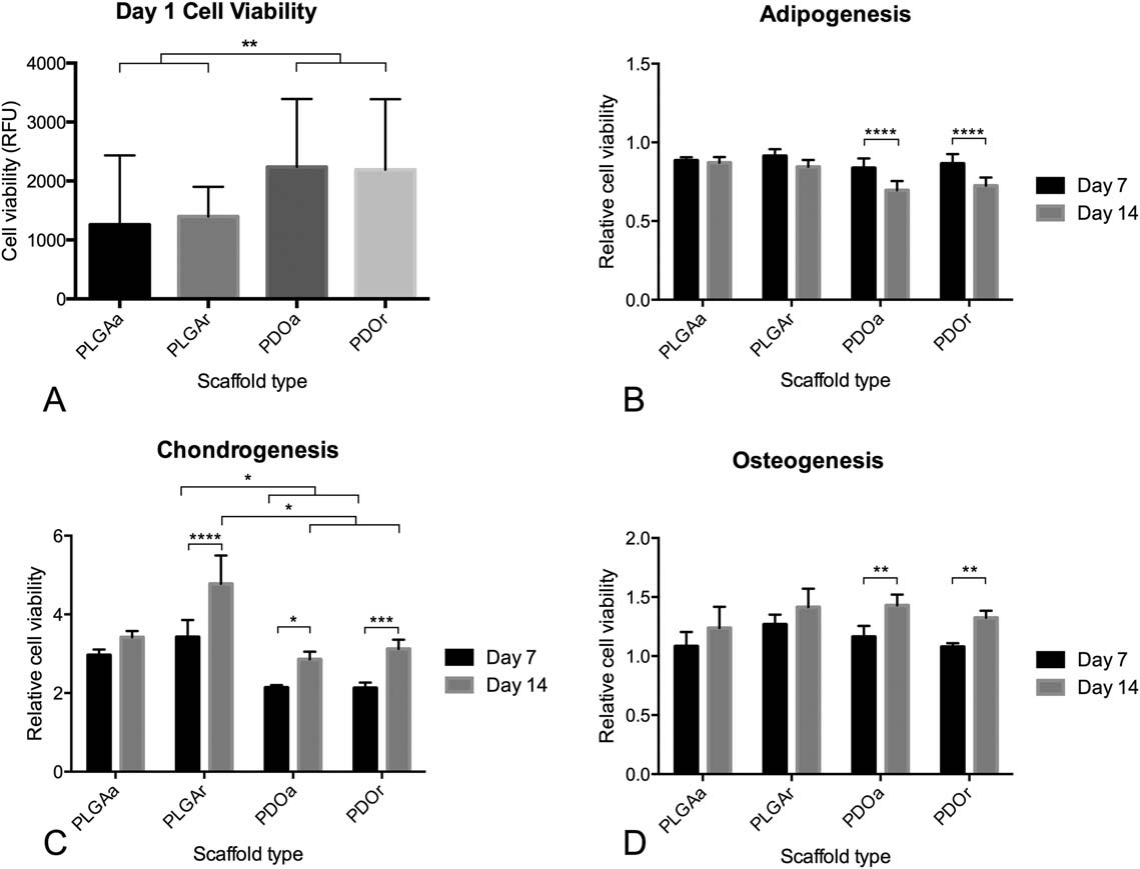
Cell viability of chondrogenic but not adipogenic and osteogenic cells was influenced by scaffold material. (A) Initial cell attachment of MSCs on scaffolds was assessed by AlamarBlue at day 1 after seeding, RFU = relative fluorescent units. Cell viability of adipogenic (B), chondrogenic (C), and osteogenic (D) MSCs was measured at days 7 and 14 after seeding (*n* = 3 MSC donors)

Cell viability on day 1 was measured to assess initial attachment of MSCs to the four scaffold types. Initial attachment was significantly higher on PDO compared with PLGA, revealing a greater initial cell attachment to PDO (*p* < 0.0001). There was no significant difference in cell viability between aligned and randomly orientated scaffolds for each material type [Fig. 4(A)].

Cell viability of chondrogenic MSCs at day 14 was higher on PLGAr compared with, PDOa (1.67‐fold, *p* = 0.003) and PDOr (1.53‐fold, *p* < 0.0001). Cells grown on PLGAa showed a showed a similar trend of higher cell viability on day 14 compared with PDO but did not reach statistical significance. There was a significant increase in chondrogenic MSC viability on PLGAr, PDOa, and PDOr between day 7 and day 14. In contrast, between day 7 and 14, cells cultured in adipogenic inducing medium showed a significant decrease in relative cell viability on PDO scaffolds [*p* < 0.0001; Fig. 4(B)], but no change in relative cell viability on PLGA scaffolds. Osteogenic MSCs cultured on PDO showed a significant increase in cell viability between days 7 and 14 [*p* < 0.001; Fig. 4(C)] but no statistically significant increase was seen for cells grown on PLGA scaffolds.

### Gene expression

**Figure 5:**
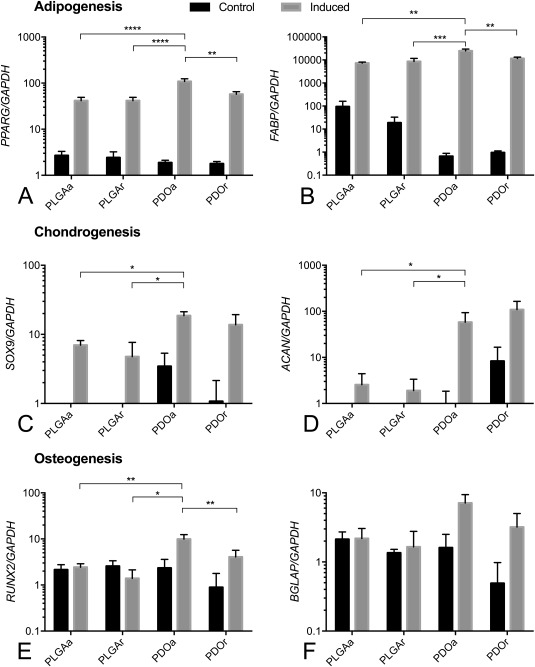
PDO scaffolds support highest adipogenic, chondrogenic, and osteogenic differentiation. Relative expression of (A, B) adipogenic, (C, D) Chondrogenic, and (E, F) osteogenic genes against *GAPDH* reference gene. *n* = 3 MSC donors.

Real‐time qPCR was used to assess the relative expression of lineage‐specific genes on different scaffold types. *PPARG* and *FABP* expression were increased in adipogenic media on all scaffolds tested, suggestive of successful adipogenic differentiation [Fig. 5(A,B)]. *PPARG* expression was highest in iPDOa scaffolds compared with iPLGAa (2.84‐fold, *p* < 0.0001), iPLGAr (2.62‐fold, *p* < 0.0001), and iPDOr (1.89‐fold, *p* = 0.002). *FABP* expression was also highest on iPDOa compared with iPLGAa (3.39‐fold, *p* = 0.01), iPLGAr (2.90‐fold, *p* = 0.0004), and iPDOr (2.14‐fold, *p* = 0.004).

Expression of chondrogenic genes *SOX9* and *ACAN* was significantly higher in MSCs cultured in chondrogenic compared with non‐inducing basal media [Fig. 5(C,D)]. Expression of *SOX9* was significantly higher in cells cultured on iPDOa scaffolds compared with iPLGAa and iPLGAr with cells isolated from iPDOr scaffolds showing a similar relative pattern of expression. *ACAN* expression was significantly higher on iPDOr compared with iPLGAa (2.68‐fold, *p* = 0.0431) and iPLGAr (3.92‐fold, *p* = 0.0101) scaffolds and those on iPDOa showed a similar trend. There was no significant difference in *SOX9* or *ACAN* expression between cells isolated from iPDOa and iPDOr scaffolds.

Expression of the osteogenic genes *RUNX2* and *BGLAP* was consistently increased in cells cultured in inducing medium on PDO but not PLGA scaffolds. Expression of *RUNX2* in osteogenic MSCs was 4.08‐fold (*p* = 0.0070) higher on iPDOa compared with iPLGAa scaffolds and 4.76‐fold higher on iPDOa compared with IPLGAr scaffolds [Fig. 5(E)]. There was no significant difference in *RUNX2* expression between osteogenic cells grown on iPDOa versus iPDOr, although there was a trend for increased *RUNX2* expression on iPDOa. Expression of *BGLAP* in osteogenic cultures followed a similar pattern to RUNX2 but did not reach statistical significance [Fig. 5(F)].

## DISCUSSION

Facilitating the differentiation of MSCs down adipogenic, chondrogenic, or osteogenic pathways using a mimetic scaffold has long been desirable. It is well established that the chemistry and morphology of scaffolds influences the propensity for cells to undergo differentiation.^13^, ^22^, ^23^, ^24^ Thus, investigating these variables and presenting direct comparisons of different polymers and morphologies is highly instructive for those wishing to design effective scaffolds. This work assessed how polymer chemistry and the fiber orientation of electrospun scaffolds affect adipogenic, chondrogenic, and osteogenic differentiation of MSCs. To our knowledge, the effect of PLGA and PDO electrospun scaffolds on multilineage MSC differentiation has not been directly compared. Our results show that both scaffold morphology and chemistry can influence adipogenic, chondrogenic, and osteogenic differentiation of MSCs.

### Adipogenic differentiation was best facilitated by aligned PDO scaffolds

Scanning electron microscopy showed greater adipogenic cell coverage on PDO compared with PLGA and thus may support better maintenance of adipocyte cell attachment and viability and thus successful incorporation following implantation.^25^ Relative cell viability at day 14 compared with day 7 was decreased on PDO scaffolds suggesting successful initiation of adipogenesis. This reduction in alamarBlue metabolic activity readings of adipogenic MSCs cultured on PDO may reflect a reduction in cell metabolic activity and proliferation that would be typical of cells that have differentiated down an adipogenic cell line, and previous studies have shown that adipogenic medium inhibits the proliferative capacity of MSCs on plastic by 50%.^26^ Finally, adipogenic gene expression was highest on aligned PDO suggesting enhanced adipogenesis. Therefore, electrospun PDO oriented in an aligned pattern is the most suitable of those tested for adipogenic differentiation of MSCs for tissue repair.

This supports previous work purporting the use of PDO scaffolds for adipose stem cell culture.^27^ Enhanced differentiation of mesenchymal stem cells on aligned over randomly orientated electrospun scaffolds is also supported on electrospun polycaprolactone scaffolds.^28^ However, it has been shown that adipogenic gene expression (*PPARG*) is lower on aligned compared with randomly orientated fibers of electrospun poly(3‐hydroxybutyrate‐co‐3‐hydroxyhexanoate scaffolds.^29^ This difference was seen when cultured in non‐inducing but not inducing medium and suggests that fiber orientation‐effects on adipogenic differentiation are influenced by the biological milieu. Although a rounder cell shape has been associated with increased propensity to undergo adipogenesis, our results have shown decreased adipogenic gene expression on iPLGAr despite the rounded morphology of cells.^30^ This may suggest that inhibition of adipogenesis is occurring through other mechanisms relating to the material properties of the scaffold. Mechanical properties of an environment may have a more important effect on MSC differentiation. It has been shown that increased matrix rigidity, and cyclic stretching of MSCs inhibits apdipogenic differentiation.^31^, ^32^ Therefore, previous studies showing that PDO is less stiff than PLGA suggests that the differences in adipogenic differentiation between these two polymers is due to the mechanical environment provided by the material, not the fiber alignment itself.^33^, ^34^, ^35^ An interesting application of these findings may be the design and testing of an electrospun PDO prototype more closely mimicking the gross structure and porosity of native adipose tissue.

### For chondrogenic differentiation, PDO scaffolds produced the most promising results

MSCs cultured in chondrogenic medium showed the highest relative cell viability on PLGA. Chondrogenesis of MSCs has been linked to low initial respiratory rates resulting from a shift to anaerobic glycolysis.^36^ Therefore, the relative increase in cell viability on PLGA could indicate a failure of chondrogenesis, suggesting that PDO supports higher chondrogenic cell growth and differentiation. This is supported by gene expression results, in which both *SOX9* and *ACAN* were more highly expressed on PDO scaffolds compared with PLGA. Furthermore close cell‐cell contact is essential for successful induction of chondrogenesis of MSCs and the low cell viability on day 1 of MSCs cultured on PLGA suggests reduced cell attachment and thus limited propensity for chondrogenic induction.^37^ In addition, it has been suggested that stiffness of the scaffold material may affect differentiation of MSCs by influencing mechanical tension of the cytoskeleton.^38^ In particular softer matrices have been found to preferentially induce chondrogenesis of MSCs. Previous reports of the mechanical properties of electrospun PDO and PLGA scaffolds has shown that PDO is less stiff (i.e., more elastic) than PLGA, and that electrospun PDO possesses comparable mechanical properties to collagen and elastin, the main structural components of the native cartilage ECM.^33^, ^34^, ^35^ This lends further significance to the apparent enhancement of chondrogenesis on PDO compared with PLGA scaffolds as suggested by gene expression results.

Chondrogenic cells have typically been associated with a more rounded cell shape, and it has previously been shown that a random mesh of fibers more closely resemble cartilage ECM, whilst aligned fibers promoted fibrous over cartilaginous differentiation of MSCs.^33^, ^39^ Our data does not, however, indicate a strong influence of random versus aligned fiber orientation on chondrogenic differentiation. Increased nodule formation on PDOr may suggest enhanced chondrogenesis following initial induction of *SOX9* and direction toward the chondrogenic lineage. However, further dissection of cell and tissue properties following growth on PDO matrices of defined and expanded fiber orientations is necessary.

### Osteogenic differentiation was favored on aligned PDO

Significantly higher expression of *RUNX2* on aligned PDO compared with PLGA suggests that PDOa facilitated higher osteogenic differentiation. The lower osteogenic differentiation on PLGA compared with PDO scaffolds may be due to the degradation products of PLGA inhibiting osteogenic induction. Previous work has shown that the degradation products lactic and glycolic acid cause a decrease in osteoblast differentiation and that the highest degradation rate of PLGA scaffolds (75%lactic, 25%glycolic acid) (as used in our experiments) occurs in the first 14 days of culture.^40^, ^41^ It has been reported that osteogenic differentiation does not result in a change in oxygen consumption of cells and, therefore, metabolic readouts of cell viability.^36^ In support of this, our data shows similar metabolic cell viability across all scaffold types, suggesting that these viability assays cannot be used to determine the most suitable scaffold material for osteogenesis, but rather to monitor for possible toxic degradation products. The higher osteogenic differentiation on aligned PDO compared with all other scaffold types supports previous work showing higher osteogenic differentiation on aligned relative to randomly orientated PLGA‐based electrospun scaffolds.^42^

In conclusion, PDO scaffolds supported better growth and differentiation of MSCs into adipocytes, chondrocytes, and osteoblasts. In comparison to PLGA, PDO may provide a better substratum for cells *in vitro*. Possible explanations to for this may be favorable mechanical properties, morphology, degradation rate, or degradation products. Although scaffold chemistry had the most pronounced effect on the differentiation capacity of MSCs, our data also suggests a role for scaffold fiber orientation. Specifically, aligned scaffolds supported higher adipogenic and osteogenic differentiation, but random scaffolds favored chondrogenesis. This is most likely to be due to their effect on cell morphology, which has been shown to directly influence osteogenic versus adipogenic differentiation.^30^ Producing scaffolds that significantly enhance differentiation toward one lineage whilst inhibiting that toward others could prevent failure of generated tissue due to fibrosis, unwanted fatty tissue formation or ossification.^43^, ^44^, ^45^

In tendon healing, tenogenic capacity has been shown to be influenced by scaffold fiber diameter.^46^ Changes in fiber diameter may impact the adhesion of cells to both the scaffold and each other, driving changes in cellular morphology and differentiation. The fiber diameter of PDOa in our study was lower than that of PDOr [Table 1, Fig. 1(B)]. There was no significant difference in pore size between scaffold types. It is possible to speculate that the increased adipogenic and osteogenic differentiation on PDOa may be a consequence of favorable changes to cell morphology via microarchitectural changes from lower fiber diameters and aligned fibers promoting differentiation toward this lineage. The fiber diameter of the two aligned scaffolds (PLGA and PDO) were also similar, but PLGAa scaffolds did not promote successful differentiation reinforcing the fundamental importance of scaffold chemistry.

This study compares scaffold chemistry and alignment on chondrogenic, adipogenic, and osteogenic differentiation. However, the work is subject to a number of limitations including the *in vitro* setting of comparisons. We have investigated two of the most prominent polymers in tissue engineering but we cannot rule out the potential for other polymers, or different percentages of PDO and PLGA to more strongly influence differentiation. Furthermore, the fiber diameter in our study varied between aligned and random scaffolds and is likely to have effects on cell phenotype independent of fiber orientation. Future work should focus on this characteristic rather than the broad range of diameters represented by the fibers in our study.

Overall, our findings suggests that *in vitro*, electrospun PDO was superior to electrospun PLGA as a substrate for MSCs differentiation into three lineages, and provides strong supporting evidence for the continued investigation of the use of an electrospun PDO scaffold for tissue repair and regeneration. Moreover, delineation of the fiber pattern (both fiber diameter and orientation) also influenced the differentiation of MSCs. These findings merit further exploration to identify the exploitable mechanisms that underlie these cell–scaffold interactions, with the ultimate goal to design a smart material that promotes the differentiation of resident stem cells without the need for *ex vivo* cell culture.

**Ethical approval**: All cells used for this work were purchased from Lonza and no institution or study specific ethical approval was therefore necessary for this study.
